# New Insights into Skeletal Muscle Metabolism in Pathological and Physiological Conditions: A Multidisciplinary and Translational Approach

**DOI:** 10.3390/biology15131052

**Published:** 2026-07-01

**Authors:** Cristina Antinozzi, Paolo Sgrò, Luigi Di Luigi

**Affiliations:** Unit of Endocrinology, Department of Movement, Human and Health Sciences, University of Rome Foro Italico, 00135 Rome, Italy; paolo.sgro@uniroma4.it (P.S.); luigi.diluigi@uniroma4.it (L.D.L.)

Skeletal muscle represents not only the largest component of our total body mass but also functions as an endocrine and metabolic organ. Beyond its role in locomotion, it is involved in energy homeostasis, hormone balance, and systemic inflammatory responses [1]. Thus, any condition that impairs skeletal muscle health could influence longevity and give rise to debilitating disorders, including muscular dystrophies, sarcopenia, cancer cachexia, and metabolic syndromes [2].

This Special Issue of *Biology*, entitled “New Insights into Skeletal Muscle Metabolism in Pathological and Physiological Conditions”, comprises twelve original contributions, including research articles and reviews, aimed at elucidating the cellular, molecular and epigenetic mechanisms underlying muscle physiology, while highlighting novel therapeutic and preventive strategies.

Advancements in multi-omics technologies have facilitated the mapping of skeletal muscle responses to metabolic stress. In their study, Cui and colleagues investigated the significant effects of prolonged starvation on gastrocnemius muscle remodelling [3]. The authors identified lysine β-hydroxybutyrylation (Kbhb) as a responsive post-translational modification involved in enzymatic activity reprogramming and cellular energy pathways. Focusing on age-related disorders, Yan et al. combined multi-omics network analysis with machine learning algorithms to identify SLC25A12 and PABPC4 as key diagnostic biomarkers in sarcopenia, thereby establishing a novel connection between copper-dependent regulated cell death (cuproptosis) and senile muscle atrophy [4]. In the context of oncological care, Verdi and colleagues reviewed the pathophysiological networks of cancer cachexia and emphasized the potential of polyphenols derived from grape seeds to modulate NF-kB and AMPK pathways.

In their study, Wilkinson and his team explored the underlying mechanisms of vascular-related myopathies, with a particular focus on the role of ferroptosis, lipid peroxidation, and autophagy in peripheral artery disease (PAD) myopathy. Their findings have significant implications for the development of targeted antioxidant therapies [5].

In recent years, there has been an increased emphasis on the rigorous evaluation of sex-specific differences in the context of modern biological research. Two studies conducted by Sgrò and colleagues have provided novel insights into the intrinsic sexual dimorphism of human primary skeletal muscle cells (46XX and 46XY) exposed to testosterone. The findings of the study demonstrate that sex chromosomes exert a direct influence on muscle steroidogenesis, hormone homeostasis, and myogenesis. The investigation reveals a preferential activation of the MAPK pathway in female cells as opposed to the PI3K/AKT pathway in male cells. These insights emphasize the pressing need for gender-specific precision medicine in the study of myopathies and muscle regeneration [6,7].

It is evident that physical exercise and nutritional interventions are foundational components for muscle development and the prevention of disease. Li, Zhu, and Yan utilized single-cell transcriptomics to establish the Fos gene as a pivotal regulator of skeletal muscle adaptation to long-term aerobic exercise, thereby demonstrating how its downregulation alleviates inflammation and enhances satellite cell-mediated muscle remodelling [8]. In the domain of pharmacological research, Ghanemi et al. have demonstrated that a single injection of SPARC (secreted protein acidic and rich in cysteine) can acutely replicate the molecular benefits of physical exercise on glucose metabolism and mitochondrial biogenesis. This finding presents a promising therapeutic avenue for patients who are immobilized or elderly [9].

In sports nutrition and performance optimization, Lin and colleagues validated the efficacy of caffeine supplementation (alone or combined with carbohydrate mouth rinsing) in suppressing fatigue and extending pulling duration in elite indoor tug-of-war athletes [10]. A study by García-Giménez et al. monitored athletes during a multi-stage Ultra-Trail event, revealing that while ad libitum hydration mitigates the risk of exercise-associated hyponatraemia, it does not fully prevent early dehydration or the severe muscle and liver damage induced by extreme exertional stress [11]. This highlights the necessity of personalized hydration and recovery protocols.

This Special Issue also includes a comparative perspective by Gundersen and Anas on skeletal muscle programming in ruminants. The review details how maternal nutrition during gestation profoundly shapes fetal myogenesis, satellite cell dynamics, and the expression of myogenic regulatory factors (MYF5, MYOD1, MYOG). This is a subject that bridges basic developmental biology with the sustainability of livestock production and the quality of meat [12].

In conclusion, the works published in this Special Issue have two principal themes. Firstly, they expand the boundaries of our understanding of muscle metabolism. Secondly, they highlight the powerful convergence of diverse disciplines, including bioinformatics, molecular biology, sports medicine and animal science. It is evident, therefore, that the present collection represents a comprehensive and state-of-the-art reference which will inevitably stimulate future research in the field of muscle health and disease.
